# Analysis of Clinical Pattern of Musculoskeletal Disorders in the Cervical and Cervico—Thoracic Regions of the Spine

**DOI:** 10.3390/jcm13030840

**Published:** 2024-02-01

**Authors:** Gabriela Figas, Joanna Kostka, Małgorzata Pikala, Jolanta Ewa Kujawa, Tomasz Adamczewski

**Affiliations:** 1 Clinic of Medical Rehabilitation, Medical University of Lodz, 92-213 Lodz, Poland; gabriela.figas@umed.lodz.pl (G.F.); jolanta.kujawa@umed.lodz.pl (J.E.K.); tomasz.adamczewski@umed.lodz.pl (T.A.); 2 Department of Gerontology, Medical University of Lodz, 93-113 Lodz, Poland; 3 Department of Epidemiology and Biostatistics, Medical University of Lodz, 90-752 Lodz, Poland; malgorzata.pikala@umed.lodz.pl

**Keywords:** cervical spine, musculoskeletal disorders, manual assessment, cervical spine disorders

## Abstract

**Background:** Cervical spine disorders (CSDs) are a common cause of neck pain. Proper diagnosis is of great importance in planning the management of a patient with neck pain. Hence, the aim of this study is to provide an overview of the clinical pattern of early-stage functional disorders affecting the cervical and cervico-thoracic regions of the spine, considering the age and sex of the subjects. **Methods:** Two hundred adult volunteers were included in the study. Manual examination of segments C0/C1-Th3/Th4 was performed according to the methodology of the Katenborn–Evjenth manual therapy concept and the spine curvatures were assessed (cervical lordosis and thoracic kyphosis). **Results:** The most common restricted movement was lateral flexion to the left, and the least disturbed movement were observed in the sagittal plane (flexion and extension). The most affected segment was C7/Th1 (71.5% participants had problems in this segment), and the least affected segment was Th3/Th4 (69.5% participants had no mobility disorders in this segment). The number of disturbed segments did not differ between men and women (*p* > 0.05), but increased with age (r = 0.14, *p* = 0.04). **Conclusions:** Cervical mobility in adult population is frequently restricted. The number of affected segments increased with age and was not sex-dependent.

## 1. Introduction

Cervical spine disorders (CSDs) are a common cause of neck pain and the fourth cause of disability worldwide [1,2,3,4].They mostly affect female adults aged 45–54 and males aged 45–59 [3], with prevalence ranging between 5.9% and 38.7% [2]. The incidence increases with age [1].

Neck pain is a multifactorial disease contributed by many risk factors. Hence, setting an early diagnosis is vital as it might help to provide patients with immediate care [5]. Scientific studies have shown a connection between shorter neck pain duration and better long-term outcomes [2,6,7]. Clinical outcomes suggest that treatment initiated three months later might turn out much less successful [2].

Neck pain can be classified on the basis of pain mechanism (specific, neuropathic, nonspecific), pain pattern (single episode, recurrent, persistent), and duration of symptoms (acute, subacute, chronic) [3]. Neck pain usually mostly subsides within 2 months following the appearance of the first symptoms. However, many patients suffer from recurrence and the pain turns into persistent neck pain [2].

Incorrect head and neck posture has long been correlated with chronic musculoskeletal pain. Forward head posture (FHP), being poor posture in the sagittal plane, is one of the most widely reported in literature [8]. It has been indicated that improper postures may contribute to the onset and persistence of neck pain syndrome with further loss of cervical spine extension [9]. The problem is related to muscle imbalance and may affect the sense of the position of the neck structures, proprioception disorders as well as dysfunction in the neuromuscular system, and changes in the function of mechanoreceptors [10]. Researchers also highlight the problem of trigeminal sensitization associated with musculoskeletal dysfunctions, which may result in an increase in the frequency and severity of migraine pain [11].

An important element of a patient examination is the assessment of the occurrence of musculoskeletal disorders in the form of limited mobility or increased muscle and/or ligament tension within the examined movement segment. The examination can be carried out by a physiotherapist or by other medical specialists in the manual examination in one of the concepts of manual therapy (e.g., the Kaltenborn–Evjenth concept or Maitland concept) [12,13]. According to Clinical Practice Guidelines Linked to the International Classification of Functioning, Disability and Health from the Orthopedic Section of the American Physical Therapy Association regarding neck pain, segmental mobility testing is one of tools that clinicians should use when evaluating patients with neck pain [14].

Every functional movement of the head and neck involves a combined work of the cervical part of the spine as well as the cervico-thoracic junction. Thus, the functional part of the cervical spine consists of cervical and upper thoracic motion segments (C0/C1-Th3/Th4). According to the Maitland concept, the cervical spine is divided into the upper part (C0/C1-C1/C2), middle part (C2/C3-C4/C5), and lower part (C5/C6-Th3/Th4) [15].

The examination of the cervical spine from a biomechanical perspective deserves special attention. Due to the anatomical structure of the cervical spine, as well as the presence of cranio-cervical and cervico-thoracic transitions, we can distinguish the so-called transition regions where mobile segments are adjacent to less mobile segments. In the transition regions, the mechanical stress to which the surrounding structures are subjected is greater than for two adjacent segments with similar mobility. In the case of the cervical spine, the transitional regions include the craniocervical junction (the skull meets the mobile cervical spine here), the C2/C3 segment (the first segment with the disc, much more restricted mobility in the direction of rotation compared to the C1/C2 segment), and segment C7/Th1 (the lordosis of the cervical spine reverses into kyphosis of the thoracic spine). This condition results in increased biomechanical stress observed at this junction [16].

Most guidelines for the diagnosis and medical management of patients with neck pain recommend a combination of manual therapy, exercise, and education as the preferred forms of evidence-based therapy [17]. That is why setting a functional diagnosis is of great importance in planning the management of a patient with neck pain.

There are no literary reports on problems associated with segmental disorders in relation to the cervical and cervico-thoracic parts of the spine in early stages, and as such occur before structural changes. Moreover, at the onset of the disease, patients often experience only minor pain symptoms and minor functional limitations [18]. We did not find any studies assessing clinical patterns of occurrence of functional disorders of the cervical spine. The studies we found on this topic tend to focus on the mobility of single segments, most often the C7/Th1 junction [19]. Identification of these patterns could contribute to a better understanding of specific mechanisms leading to dysfunctions in the cervical spine. Such information could also contribute to the invention of preventive programs aimed at the most common problems related to cervical spine dysfunction. Hence, the aim of this study is to provide an overview of the clinical pattern (the most often and the least often restricted intersegmental movements as well as the most and the least affected segments) of early-stage functional disorders affecting the cervical and cervico-thoracic regions of the spine, and to assess whether other factors (age and sex) influence the incidence of intersegmental movement disorders.

## 2. Materials and Methods

### 2.1. Participants

The study group included 200 volunteers, with and without neck pain, selected from among the participants qualified for the first stage of the VRneck SOLUTION project—a medical experiment entitled “Evaluation of the usefulness of available 3D technologies for visualization of functional motor tasks in the cervical spine”. Inclusion criteria included the age between 18 and 80 years. Exclusion criteria were any past major cardiological, endocrine, psychiatric, neurological, cancerous, orthopedic, or inflammatory diseases, as well as any other condition that could affect functioning of the spine of the study area, including severe structural changes and instability (assessed with X-rays and during a physical examination). The full list of exclusion criteria is available in Appendix A. All participants agreed and signed a written consent to take part in this study.

The study was approved by the Bioethics Committee of the Medical University of Lodz (RNN/115/21/KE, approved 11 May 2021) and was conducted in accordance with the Declaration of Helsinki for research involving human subjects.

### 2.2. Protocol

**Figure 1:**
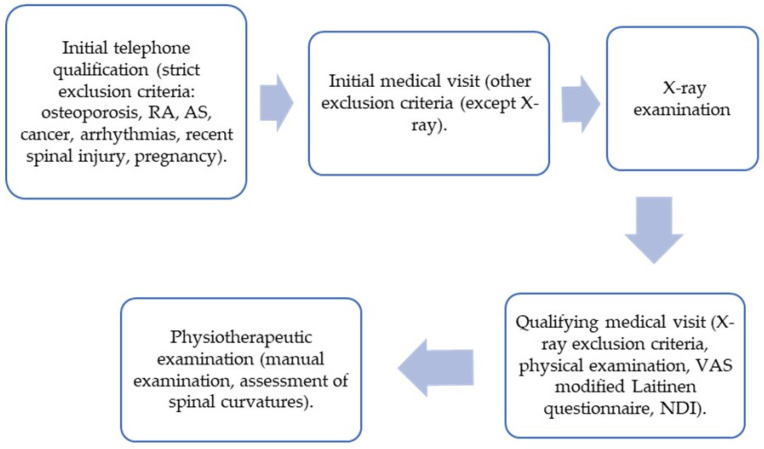
Study protocol. RA—rheumatoid arthritis; AS—ankylosing spondylitis; VAS—Visual Analogue Scale; and NDI—Neck Disability Index questionnaire.

Volunteers were recruited through correspondence with workplaces, universities and universities of the third age, and through information put in the press and social media. The patients were diagnosed and qualified for the study on the basis of an initial telephone call, an initial medical visit, an X-ray examination, a qualifying medical visit, and a physiotherapeutic examination. The patient’s diagnostic path is presented in Figure 1.

#### 2.2.1. Manual Examination

Manual examination included the assessment of the segmental mobility of the cervical and cervico-thoracic regions of the spine (segments C0/C1-Th3/Th4) according to the methodology of the Kaltenborn–Evjenth Orthopedic Manipulative Therapy (KEOMT) [20]. The examination was performed by physiotherapists specialized in manual therapy for specific movements taking place in each spinal segment. It started with an assessment of the upper segments and finished with an assessment of the lower segments. Possible observations for each movement at the segment level included: unrestricted movement, restricted movement, and no movement at all or “no observation”. The patient was in a sitting position during the examination.

##### Patient Examination Methodology—General Principles

− C0/C1—physiotherapist assesses movements taking place in this segment (flexion, extension, flexion to the left with rotation to the right, and flexion to the right with rotation to the left). The physiotherapist passively moves the head with one hand while the second hand palpates the space between the transverse process of C1 and mastoid process of the temporal bone. The examination is carried out on both sides.
− C1/C2—physiotherapist assesses movements taking place in this segment (flexion, extension, rotation to the left, and rotation to the right). The physiotherapist passively moves the head and neck with one hand while the second hand palpates the space between the vertebral arc of C1 and the spinous process of C2.
− C2/C3-Th3/Th4—physiotherapist assesses movements taking place in each segment (flexion, extension, lateral flexion to the left, lateral flexion to the right, rotation to the left, and rotation to the right). The physiotherapist passively moves the head and neck with one hand while the second hand palpates the space between the vertebral spinous processes of the tested segment.

##### Interpretation

Assessment of the movement of topographic points of a particular segment allows to determine whether the patient demonstrates limited cervical mobility. When the answer was “unrestricted movement”, the movement in physiotherapeutic assessment was normal in comparison to the upper/lower segment or the other side. The segment was considered disturbed when the answer was “restricted movement” or “no movement”. “No observation” means that the physiotherapist was not able to examine movement due to, for example, the increased muscle tone.

#### 2.2.2. Assessment of Spinal Curvature

During the examination, the physiotherapist assessed the position of the head and neck in relation to the shoulder girdle. Spinal curvatures of cervical lordosis and thoracic kyphosis were assessed. The patient was examined in a standing position, sideways to the physiotherapist. Possible observations included preserved/abolished/deepened cervical lordosis and preserved/abolished/deepened thoracic kyphosis.

#### 2.2.3. Assessment of Pain Intensity

The visual analog scale (VAS) and a modified Laitinen questionnaire were used to assess the intensity of pain of the studied participants.

VAS is a self-report scale consisting of a horizontal or vertical line, usually 10 cm (100 mm) long, with clearly marked starting points (0—no pain) and ending points (10—unbearable pain) [21]. The examiner asked the patient to mark a line at the point that best related to his current pain intensity [21,22]. Results are presented in the point form and range from 0 to 10.

The modified Laitinen Questionnaire includes questions about pain intensity, frequency of pain, use of painkillers, and limitation of physical activity. Each pain indicator is rated on a five-point scale, ranging from 0 to 4 points. The total score in these four domains ranges from 0 to 16 points, where a lower score indicates a better functional status of the patient [23,24].

#### 2.2.4. Assessment of Neck Pain-Related Disability

The NDI questionnaire allows to assess disability caused by pain in the cervical spine and is one of the most frequently used indices to assess neck pain in clinical trials [25,26].

The scale includes 10 categories of questions assessing the ability of performing everyday activities. Self-assessment includes the severity of pain, independence in everyday activities, lifting objects, reading, headaches, concentration, work, driving, sleep, and active rest in relation to ailments in the cervical spine. The obtained result is given in numerical values (0–50) or in percentage units. The higher a patient’s score, the greater his or her level of disability [27].

#### 2.2.5. Statistical Analysis

In the statistical analysis, categorical variables were described with frequencies and corresponding percentages. Arithmetic means and standard deviations were calculated for quantitative variables. The statistical significance of the differences between women and men was calculated using the Mann–Whitney U test. The Pearson’s correlation coefficient r was used to assess the correlation between the number of disturbed spinal segments and age. The coefficient of determination R^2^ was added as an effect measure, calculated as the square of the Pearson correlation r. The coefficient of determination is the proportion of the variation in the dependent variable that is predictable from the independent variable. In the case of paired data, this is a measure of the proportion of variance shared by the two variables and varies from 0 to 100%. The statistical analysis was made using STATISTICA version 13.3. The *p* ≤ 0.05 level was adopted as significant.

## 3. Results

### 3.1. Characteristics of the Study Group

The study group included 200 participants, both sexes (135 females and 65 males), aged 19–78, with pain ranging from no pain to severe pain problems and without severe neck disability (NDI 0–26 points). The number of segments with movement restrictions was 0–11 (Table 1).

### 3.2. Functional Disorders Observed in Segments C0/C1-Th3/Th4

Types of mobility restrictions for individual segments are shown in Table 2. The most common restricted movement was lateral flexion to the left (7 out of 11 segments for simple movements and additionally combined movement with rotation to the right in the C0/C1 segment). The least disturbed movements were noted in the sagittal plane for flexion and extension (6 and 5 out of 11 segments, respectively).

The largest number of disorders, i.e., in 71.5% of the participants, was confirmed in the C7/Th1 segment, whereas the Th3/Th4 segment was the least disturbed. No mobility disorders in the above segment were confirmed in 69.5% of the examined participants. The list of segments with and without mobility restrictions is shown in Table 3.

### 3.3. Functional Disorders in Relation to the Upper, Middle, and Lower Cervical Spine

The authors made an analysis of functional disorders in relation to the incidence of disorders.

#### 3.3.1. Upper Cervical Spine (C0/C1-C1/C2)—2 Segments

Overall, 50.5% of participants did not report any disorders in this region, and 49.5% of participants reported disorders in one or more segments (21% had disorders in 1 out of 2 segments, and 28.5% had disorders in all 2 segments). The average number of segments with movement restrictions for this part of the cervical spine was 0.78 ± 0.86.

#### 3.3.2. Middle Cervical Spine (C2/C3-C4/C5)—3 Segments

A total of 18.5% of participants did not report any disorders in this region, whereas 81.5% of participants reported disorders in one or more segments (17.5% had disorders in 1 out of 3 segments, 30% had disorders in 2 out of 3 segments, and 34% had disorders in all 3 segments).

The average number of segments with movement restrictions for this part of the cervical spine was 1.80 ± 1.10.

#### 3.3.3. Lower Cervical Spine (C5/C6-Th3/Th4)—6 Segments

Overall, 10.5% of participants had no disorders in this region, whereas 89.5% of them had disorders in one or more segments (14%, 18.5%, 17.5%, 14%, and 7.5% of participants reported disorders in 1 out of 6 segments, 2 out of 6, 3 out of 6, 4 out of 6, 5 out of 6 segments, respectively; 18% of participants reported disorders in all 6 segments). The average number of segments with movement restrictions for this part of the cervical spine was 3.05 ± 1.94.

### 3.4. Functional Disorders in Relation to Sex and Age

The authors made an analysis of the number of disturbed segments and the incidence of disorders (upper/middle/lower cervical spine) in relation to sex and age.

#### 3.4.1. Functional Disorders in Relation to Sex

The average number of segments with movement restrictions was 5.59 ± 3.08 for women and 5.71 ± 3.06 for men. An analysis of the number of disturbed segments and incidence of disorders (upper/middle/lower cervical spine) confirmed no statistically significant differences with regards to sex.

#### 3.4.2. Functional Disorders in Relation to Age

**Figure 2:**
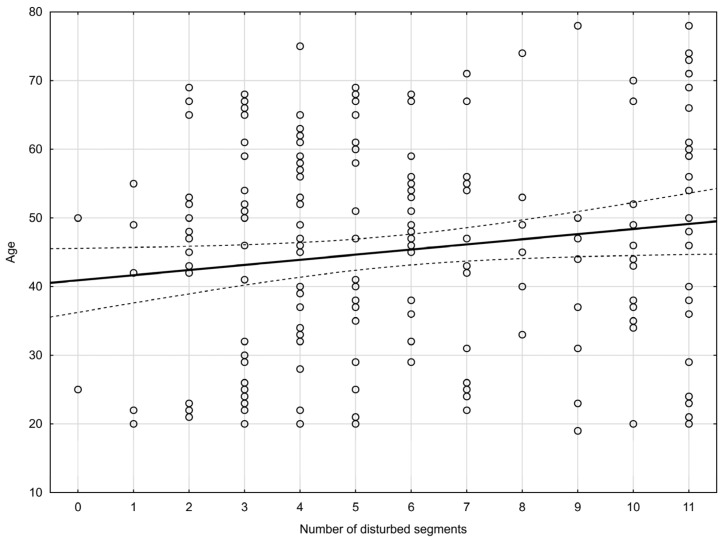
Pearson’s correlation coefficient (r = 0.14, *p* = 0.04)—incidence of disorders (number of disturbed segments) in relation to age. (solid line—regresion line, dashed line—95% confidence interval).

**Figure 3:**
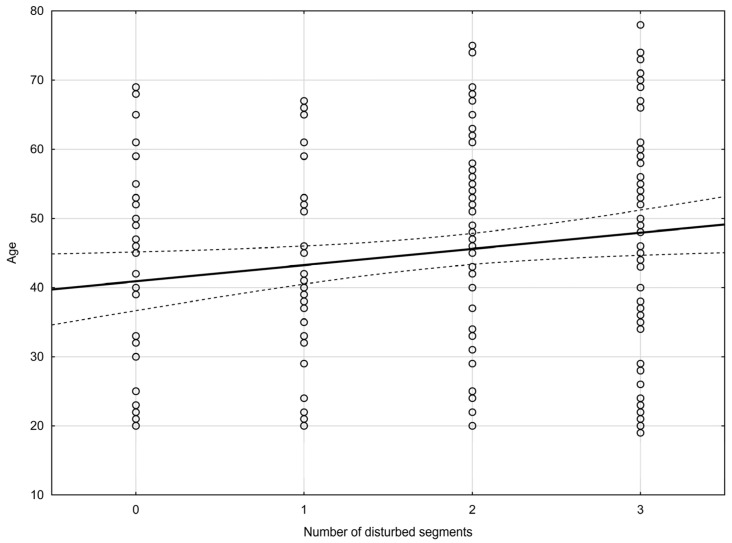
Pearson’s correlation coefficient (r = 0.16, *p* = 0.02)—incidence of disorders (number of disturbed segments) for middle part of cervical spine in relation to age. (solid line—regresion line, dashed line—95% confidence interval).

An analysis of the incidence of disorders in relation to age confirmed a correlation. The older the patient was, the greater the number of functionally disturbed segments he/she reported (the Pearson’s correlation coefficient—Figure 2). An analysis of the incidence of disorders showed a statistically significant difference for the middle part of the cervical spine (the Pearson’s correlation coefficient—Figure 3). The coefficient of determination R^2^ was added as an effect measure. No statistically significant differences were found for the upper and lower cervical spine. The results are shown in Table 4.

### 3.5. Assessment of Spinal Curvatures

An analysis of the studied population confirmed that only 38% of the surveyees had preserved cervical lordosis, 35% had abolished cervical lordosis and 27%—deepened cervical lordosis.

With regards to thoracic kyphosis, preserved curvature was observed in 46% of participants, abolished kyphosis in 37.5%, and deepened kyphosis in 16.5%.

There was no correlation between the abnormal shape of the spinal curvatures and the number of disturbed segments of the spine.

## 4. Discussion

The main goal of this study was to present the clinical pattern of early-stage functional disorders affecting the cervical and cervico-thoracic regions of the spine pain; they can foreshadow the occurrence of more serious changes in the spine and paraspinal tissues. Previous research on this subject focused mainly on the incidence of pain syndromes in this spinal region [5,28], a global range of motion assessment [29] or data were presented in smaller groups in studies on the reliability of manual examination [30].

According to our research, the incidence of intervertebral mobility restrictions in the assessed spine section is quite high. The average number of segments with movement restrictions in our patients was above 5.5 (5.63 ± 3.07). Only 2 respondents reported no movement restrictions, whereas 24 of them (12%) reported restrictions in all 11 segments. Since functional changes are not always manifested with pain, the incidence of segmental mobility disorders is higher than the frequency of neck pain reported in epidemiological studies [5,28]. Our research reveals a high frequency of functional disorders in the studied population. Hence, there is a great need to introduce preventive interventions, aiming at restoring proper biomechanical conditions in spinal joints, which will, in turn, prevent more serious dysfunctions. Structural pathology is not always a potential cause of neck pain. According to the Task Force on Neck Pain, no signs of pathology occur in the two first grades of the clinical classification system for neck pain [18]. In the initial period of the degenerative process, these changes can manifest with temporary dysfunction and include, for example, muscle strain or facet joint disorders [31]. Another problem of patients with neck pain may also be impaired proprioception, which leads to impaired sensorimotor control in the cervical part. These disturbances, secondary to neck pain, are considered a protective response limiting further stimulation of the painful tissue. In the long term, they can cause further tissue damage, aggravate pain by sensitizing the nervous systems, and promote dysfunctional movement patterns. On the other hand, segmental mobility disorders may result in the inappropriate activation of deep segmental muscles and be responsible for incorrect proprioceptive information [32].

We also made an attempt to determine which direction of movement is most often disturbed. The identification of the most characteristic clinical pattern of functional disorders of the cervical and cervico-thoracic spine may contribute to a better understanding of the mechanisms underlying the development of cervical dysfunctions, and thus to inventing better preventive and therapeutic programs. Lateral flexion to the left (7 out of 11 segments for simple movements and additionally combined movement with rotation to the right in the C0/C1 segment) turned out to be the most common restricted movement. It seems that the limitation of segmental mobility in this direction may be related to asymmetry of everyday activities, which is associated with the lateralization of the body. We did not find any studies on differences in segmental mobility for this movement (left/right flexion). In studies assessing the global range of motion of the cervical spine, the difference between the sides is usually hardly noticeable [29,33,34,35], whereas studies on muscle strength reveal a difference between the sides, where one side (usually the right one) is dominant [36].

Segmental mobility disorders in the study group most often affected the C7/Th1 segment (71.5%), and the least often the Th3/Th4 segment (30.5%). Such a high incidence of this problem affecting the cervico-thoracic junction (C7/Th1) may be due to the fact that it is a transitional part between the mobile cervical lordosis and the immobile kyphotic thoracic part [37]. These data are disturbing. In a 2-year prospective study, impaired mobility of the C7/Th1 segment was a risk factor for musculoskeletal neck-shoulder pain (the predictive value was as high as 84%). The authors suggest that the lack of synchronous mobility distribution between motion segments might be a provoking factor for pain in the neck–shoulder area [19].

The aim of our study was also to assess whether sex influences the occurrence of functional disorders. According to epidemiological studies, women are more likely to experience pain in the cervical part of the spine [5,38,39]. Despite this observation, our research revealed no differences in the number of affected segments between males and females. This may be due to the fact that in our research, we considered factors (disorders of segmental mobility) not directly influenced by psychological characteristics and associated with sensitivity. It can be also assumed that this is due to the early stage of functional disorders in the study group, in which pain symptoms do not occur or are mild. Studies show that women are characterized with a lower pain threshold and tolerance and are more likely to experience more intense pain and unpleasant stimuli [40]. Female gender is also one of risk factors of anxiety and depression [41]. Psychological factors, especially mood disorders, predispose to neck pain [5]. In a Chinese study conducted by Xu Y et al. (2020), people affected by any mental disorder (particularly mood disorders) were over two times more likely to suffer chronic back pain (neck or back) [42].

As it was expected, the number of affected segments increased with age. In accordance with our recruitment process, we excluded patients with serious structural changes. However we noted a relationship between diagnosed disorders and age. According to literature reports, structural and functional changes occur in almost all components of the musculoskeletal system (including skeletal muscles, tendons, ligaments, bones, and joint cartilage), which is a consequence of the aging process [43]. These age-related tissue changes reduce body capacity to maintain proper mobility as well as resistance to injury and overload. As a consequence of aging, the biomechanical conditions of the musculoskeletal system also deteriorate. Among others, the anterior positioning of the head increases linearly with age [44].

The cervicothoracic spinal curvature undergoes progressive change. Cranial migration of the inflection point between thoracic kyphosis and cervical lordosis and shifts in the cervical curve apex can be also observed [45]. Degenerative changes in the cervical spine are often accompanied by a shortening of the anterior or posterior vertebral column [46], which in turn results in a changed sagittal profile of the cervical spine. There are discussions in literature reports regarding the cause-and-effect relationship between spinal curvature disorders and pain in the perivertebral area. Opinions on this issue are controversial, so further in-depth research is required to determine whether, from a biomechanical point of view, the abolition of physiological cervical lordosis may cause pain in this spinal part due to muscle imbalance or kyphotic deformities [47].

### Limitation of the Study

This study has some limitations. First of all, in some studies, the repeatability of the manual examination of intersegmental movements is questioned, particularly in patients without pain [30]. However, in a study conducted by Hariharan et al. (2021) on inter-examiner reliability of physical examination procedures assessing the cervical spine, PABAK values (Prevalence And Bias Adjusted Kappa) for segmental mobility without pain provocation were moderate for mid-cervical (0.45) and low-cervical (0.53) regions [48]. According to these authors, some tests with obtained lower Kappa values may still be invaluable when making clinical decisions. Therefore, a Kappa value above 0.40 is proposed as the minimum acceptable cut-off for tests used in clinical settings. Despite unfavorable comments regarding the repeatability of manual examination, this kind of examination is often the base of manual therapy in clinical practice. Furthermore, the tests were conducted by experienced manual therapists, which affects the quality of the results [49]. Since we did not enjoy access to objective tools for assessing spinal curvatures, we had to apply only a subjective assessment method. This fact was also a limitation of the study.

We qualified people aged 18 to 78 for the study. The age range of the participants is relatively large, which is also a weakness of this study. However, we wanted to determine whether the problem of segmental mobility disorders changes with age. Finally, the study group was not large-sized. Research on a larger group of participants and focusing on possible factors determining the development of functional disorders are worth continuing.

The results of the study may be a starting point for prospective studies that will determine if and to what extent segmental mobility disorders contribute to more serious changes in the cervical and cervico-thoracic parts of the spine.

## 5. Conclusions

The frequency of cervical mobility restriction is high in the study group. Segmental mobility disorders most often affected the C7/Th1 segment. Lateral flexion to the left appeared to be the most frequently impaired direction of movement. The number of affected segments increased with age and was independent of sex.

## Figures and Tables

**Table 1:** Characteristics of the participants (n = 200).

Variable	Mean ± SD
Age (years)	45.1 ± 16.1
BMI (kg/m^2^)	25.69 ± 4.09
VAS (points)	3.1 ± 2.3
modified Laitinen questionnaire (points)	3.5 ± 2.4
NDI (points)	7.6 ± 6.1
Average number of segments with movement restrictions	5.63 ± 3.07

**Table 2:** Types of mobility restrictions for specific movements observed in segment C0/C1-Th3/Th4 in the whole sample (n = 200).

**C0/C1**						
**Mobility Restrictions**	**Flexion** **n [%]**	**Extension** **n [%]**	**Lateral Flexion to the Left with Rotation to the Right** **n [%]**		**Lateral Flexion to the Right with Rotation to the Left** **n [%]**	
Unrestricted movement	163 [81.5]	168 [84.0]	147 [73.5]		151 [75.5]	
Restricted movement	33 [16.5]	29 [14.5]	46 [23.0]		44 [22.0]	
No movement	1 [0.5]	2 [1.0]	6 [3.0]		4 [2.0]	
No observation	3 [1.5]	1 [0.5]	1 [0.5]		1 [0.5]	
**C1/C2**						
**Mobility Restrictions**	**Flexion** **n [%]**	**Extension** **n [%]**	**Rotation to the Left** **n [%]**		**Rotation to the Right** **n [%]**	
Unrestricted movement	176 [88.0]	175 [87.5]	147 [73.5]		147 [73.5]	
Restricted movement	21 [10.5]	19 [9.5]	48 [24]		47 [23.5]	
No movement	-	1 [0.5]	-		2 [1.0]	
No observation	3 [1.5]	5 [2.5]	5 [2.5]		4 [2.0]	
**C2/C3**						
**Mobility Restrictions**	**Flexion** **n [%]**	**Extension** **n [%]**	**Lateral Flexion to the Left ** **n [%]**	**Lateral Flexion to the Right ** **n [%]**	**Rotation to the Left** **n [%]**	**Rotation to the Right** **n [%]**
Unrestricted movement	174 [87.0]	178 [89.0]	122 [61.0]	140 [70.0]	149 [74.5]	156 [78.0]
Restricted movement	21 [10.5]	17 [8.5]	70 [35.0]	55 [27.5]	44 [22.0]	36 [18.0]
No movement	1 [0.5]	1 [0.5]	3 [1.5]	2 [1.0]	2 [1.0]	2 [1.0]
No observation	4 [2.0]	4 [2.0]	5 [2.5]	3 [1.5]	5 [2.5]	6 [3.0]
**C3/C4**						
**Mobility Restrictions**	**Flexion** **n [%]**	**Extension** **n [%]**	**Lateral Flexion to the Left ** **n [%]**	**Lateral Flexion to the Right ** **n [%]**	**Rotation to the Left** **n [%]**	**Rotation to the Right** **n [%]**
Unrestricted movement	178 [89.0]	184 [92.0]	118 [59.0]	139 [69.5]	146 [73.0]	154 [77.0]
Restricted movement	18 [9.0]	12 [6.0]	72 [36.0]	51 [25.5]	48 [24.0]	35 [17.5]
No movement	1 [0.5]	1 [0.5]	4 [2.0]	5 [2.5]	-	3 [1.5]
No observation	3 [1.5]	3 [1.5]	6 [3.0]	5 [2.5]	6 [3.0]	8 [4.0]
**C4/C5**						
**Mobility Restrictions**	**Flexion** **n [%]**	**Extension** **n [%]**	**Lateral Flexion to the Left ** **n [%]**	**Lateral Flexion to the Right ** **n [%]**	**Rotation to the Left** **n [%]**	**Rotation to the Right** **n [%]**
Unrestricted movement	180 [90.0]	183 [91.5]	135 [67.5]	140 [70.0]	158 [79.0]	163 [81.5]
Restricted movement	18 [9.0]	11 [5.5]	55 [27.5]	50 [25]	37 [18.5]	30 [15.0]
No movement	1 [0.5]	-	5 [2.5]	4 [2.0]	2 [1.0]	1 [0.5]
No observation	1 [0.5]	6 [3.0]	5 [2.5]	6 [3.0]	3 [1.5]	6 [3.0]
**C5/C6**						
**Mobility Restrictions**	**Flexion** **n [%]**	**Extension** **n [%]**	**Lateral Flexion to the Left ** **n [%]**	**Lateral Flexion to the Right ** **n [%]**	**Rotation to the Left** **n [%]**	**Rotation to the Right** **n [%]**
Unrestricted movement	172 [86.0]	178 [89.0]	146 [73.0]	140 [70.0]	158 [79.0]	161 [80.5]
Restricted movement	21 [10.5]	13 [6.5]	43 [21.5]	50 [25.0]	36 [18.0]	31 [15.5]
No movement	3 [1.5]	1 [0.5]	3 [1.5]	3 [1.5]	3 [1.5]	1 [0.5]
No observation	4 [2.0]	8 [4.0]	8 [4.0]	7 [3.5]	3 [1.5]	7 [3.5]
**C6/C7**						
**Mobility Restrictions**	**Flexion** **n [%]**	**Extension** **n [%]**	**Lateral Flexion to the Left ** **n [%]**	**Lateral Flexion to the Right ** **n [%]**	**Rotation to the Left** **n [%]**	**Rotation to the Right** **n [%]**
Unrestricted movement	172 [86.0]	153 [76.5]	134 [67.0]	140 [70.0]	160 [80.0]	160 [80.0]
Restricted movement	26 [13.0]	37 [18.5]	56 [28.0]	46 [23.0]	32 [16.0]	33 [16.5]
No movement	-	6 [3.0]	4 [2.0]	6 [3.0]	5 [2.5]	2 [1.0]
No observation	2 [1.0]	4 [2.0]	6 [3.0]	8 [4.0]	3 [1.5]	5 [2.5]
**C7/Th1**						
**Mobility Restrictions**	**Flexion** **n [%]**	**Extension** **n [%]**	**Lateral Flexion to the Left ** **n [%]**	**Lateral Flexion to the Right ** **n [%]**	**Rotation to the Left** **n [%]**	**Rotation to the Right** **n [%]**
Unrestricted movement	151 [75.5]	108 [54.0]	118 [59.0]	128 [64.0]	143 [71.5]	142 [71.0]
Restricted movement	40 [20.0]	72 [36.0]	62 [31.0]	49 [24.5]	45 [22.5]	48 [24.0]
No movement	3 [1.5]	18 [9.0]	11 [5.5]	15 [7.5]	9 [4.5]	7 [3.5]
No observation	6 [3.0]	2 [1.0]	9 [4.5]	8 [4.0]	3 [1.5]	3 [1.5]
**Th1/Th2**						
**Mobility Restrictions**	**Flexion** **n [%]**	**Extension** **n [%]**	**Lateral Flexion to the Left ** **n [%]**	**Lateral Flexion to the Right ** **n [%]**	**Rotation to the Left** **n [%]**	**Rotation to the Right** **n [%]**
Unrestricted movement	151 [75.5]	128 [64.0]	119 [59.5]	130 [65.0]	138 [69.0]	143 [71.5]
Restricted movement	39 [19.5]	52 [26.0]	54 [27.0]	44 [22.0]	43 [21.5]	45 [22.5]
No movement	5 [2.5]	17 [8.5]	14 [7.0]	16 [8.0]	15 [7.5]	10 [5.0]
No observation	5 [2.5]	3 [1.5]	13 [6.5]	10 [5.0]	4 [2.0]	2 [1.0]
**Th2/Th3**						
**Mobility Restrictions**	**Flexion** **n [%]**	**Extension** **n [%]**	**Lateral Flexion to the Left ** **n [%]**	**Lateral Flexion to the Right ** **n [%]**	**Rotation to the Left** **n [%]**	**Rotation to the Right** **n [%]**
Unrestricted movement	164 [82.0]	138 [69.0]	137 [68.5]	135 [67.5]	139 [69.5]	146 [73.0]
Restricted movement	24 [12.0]	38 [19.0]	32 [16.0]	32 [16.0]	40 [20.0]	39 [19.5]
No movement	6 [3.0]	18 [9.0]	24 [12.0]	23 [11.5]	16 [8.0]	10 [5.0]
No observation	6 [3.0]	6 [3.0]	7 [3.5]	10 [5.0]	5 [2.5]	5 [2.5]
**Th3/Th4**						
**Mobility Restrictions**	**Flexion** **n [%]**	**Extension** **n [%]**	**Lateral Flexion to the Left ** **n [%]**	**Lateral Flexion to the Right ** **n [%]**	**Rotation to the Left** **n [%]**	**Rotation to the Right** **n [%]**
Unrestricted movement	163 [81.5]	147 [73.5]	143 [71.5]	146 [73.0]	142 [71.0]	146 [73.0]
Restricted movement	18 [9.0]	23 [11.5]	15 [7.5]	18 [9.0]	25 [12.5]	28 [14.0]
No movement	11 [5.5]	24 [12.0]	30 [15.0]	26 [13.0]	28 [14.0]	18 [9.0]
No observation	8 [4.0]	6 [3.0]	12 [6.0]	10 [5.0]	5 [2.5]	8 [4.0]

n—number of cases.

**Table 3:** Overall movement restrictions for single segments.

Segments	Mobility Disorders n [%]	No Mobility Disorders n [%]
C0/C1	77 [38.5]	123 [61.5]
C1/C2	79 [39.5]	121 [60.5]
C2/C3	124 [62.0]	76 [38.0]
C3/C4	127 [63.5]	73 [36.5]
C4/C5	108 [54.0]	92 [46.0]
C5/C6	102 [51.0]	98 [49.0]
C6/C7	101 [50.5]	99 [49.5]
C7/Th1	143 [71.5]	57 [28.5]
Th1/Th2	120 [60.0]	80 [40.0]
Th2/Th3	83 [41.5]	117 [58.5]
Th3/Th4	61 [30.5]	139 [69.5]

n—number of cases.

**Table 4:** Pearson’s correlation coefficient for number of functionally disturbed segments in relation to age according to the incidence of disorders.

Pair of Variables	n	r	R^2^ (%)	*p*
Disturbed segments & age	200	0.14	2.02	0.04
Upper part—disturbed segments & age	200	0.06	0.34	0.41
Middle part—disturbed segments & age	200	0.160	2.58	0.02
Lower part—disturbed segments & age	200	0.11	1.15	0.13

n—number of cases.

## Data Availability

The measurement data used to support the findings of this study are available from the corresponding author upon request.

## Appendix A

The full list of exclusion criteria:

- unstable ischemic heart disease,
- acute coronary syndrome in the last three months,
- hemodynamically significant heart defects,
- arrhythmias—atrial fibrillation (paroxysmal and persistent), uncontrolled heart rhythm disturbances,
- heart failure above class II acc. NYHA,
- uncontrolled hypertension (blood pressure at visit > 150/95 mmHg,
- untreated hyperthyroidism and/or strumectomy performed within 3 months before visit 1 and/or the presence of a large thyroid goiter impairing the mobility of the neck,
- uncontrolled diabetes and/or diabetic retinopathy and/or diabetic neuropathy,
- diagnosed polyneuropathy or neuropathy,
- obesity with a BMI > 35,
- diagnosed osteoporosis and/or a history of low-energy fractures
- condition after craniocerebral injuries with hospitalization for more than 7 days, up to 6 months,
- status after neurosurgical operations in the field of the brain performed up to 12 months,
- condition after documented spinal injuries up to 6 months,
- status after spinal surgeries performed up to 12 months,
- persistent ailments after spine surgeries and/or spinal implants,
- instability of the cervical spine above 4 mm found in an X-ray examination,
- spine degenerative disease Kellgren-Lawrence-Grading-System 4 degrees found in an X-ray examination,
- cervical lordosis disorders—kyphotization found in an X-ray examination,
- fractures within the articular bodies and processes, spinous found in an X-ray examination,
- congenital defects of the spine (bone blocks, semivertebrae, Kimerle’s syndrome, other congenital defects) found in an X-ray examination,
- radiological features of osteoporosis (including fracture),
- inflammation of the shaft and disc—discitis found in an X-ray examination,
- condition after surgery with an implant or cement found in an X-ray examination,
- neoplastic changes, metastases to the bones of the spine found in an X-ray examination,
- symptomatic vertebral and basal artery insufficiency and/or a positive De Kleyn test,
- diagnosed with RA (rheumatoid arthritis),
- diagnosed AS (ankylosing spondylitis),
- diagnosed exacerbation of PsA (psoriatic arthritis) within 12 months,
- exacerbation of other systemic connective tissue diseases involving the cervical spine diagnosed within 12 months,
- past or active inflammation of the spine,
- significant mental disorders that may affect the safety or results of the experiment, taking psychoactive substances, addiction to drugs, legal highs, drugs, alcohol now and in the past,
- hemophilia and other bleeding disorders,
- patients treated with oral or parenteral anticoagulants,
- active cancer,
- condition after anti-cancer treatment including chemotherapy or radiotherapy,
- infectious diseases untreated or under treatment,
- abdominal surgeries up to 12 weeks after surgery,
- chest surgeries up to 12 weeks after surgery,
- pregnancy,
- active gastric and/or duodenal ulcer,
- expected unavailability of the patient during the experiment or up to 1 month after its completion,
- current participation in another medical experiment,
- current legal and/or insurance claims regarding the patient’s health,
- inability to assume a sitting position on your own,
- lack of cooperation on the part of the patient.
